# Graph-based feature extraction: A new proposal to study the classification of music signals outside the time-frequency domain

**DOI:** 10.1371/journal.pone.0240915

**Published:** 2020-11-12

**Authors:** Dirceu de Freitas Piedade Melo, Inacio de Sousa Fadigas, Hernane Borges de Barros Pereira

**Affiliations:** 1 Department of Mathematics of the Federal Institute of Education Science and Technology Bahia, Salvador, Brazil; 2 State University of Feira de Santana, Bahia, Brazil; 3 Computational Modeling Program, SENAI CIMATEC University Center, Salvador, Brazil; 4 University of the State of Bahia, Salvador, Brasil; Instituto Nacional de Medicina Genomica, MEXICO

## Abstract

Most feature extraction algorithms for music audio signals use Fourier transforms to obtain coefficients that describe specific aspects of music information within the sound spectrum, such as the timbral texture, tonal texture and rhythmic activity. In this paper, we introduce a new method for extracting features related to the rhythmic activity of music signals using the topological properties of a graph constructed from an audio signal. We map the local standard deviation of a music signal to a visibility graph and calculate the modularity (*Q*), the number of communities (*Nc*), the average degree (〈*k*〉), and the density (Δ) of this graph. By applying this procedure to each signal in a database of various musical genres, we detected the existence of a hierarchy of rhythmic self-similarities between musical styles given by these four network properties. Using *Q*, *Nc*, 〈*k*〉 and Δ as input attributes in a classification experiment based on supervised artificial neural networks, we obtained an accuracy higher than or equal to the beat histogram in 70% of the musical genre pairs, using only four features from the networks. Finally, when performing the attribute selection test with *Q*, *Nc*, 〈*k*〉 and Δ, along with the main signal processing field descriptors, we found that the four network properties were among the top-ranking positions given by this test.

## Intoduction

The extraordinary growth in digital music production, storage and sharing has spurred the emergence of platforms with increasingly sophisticated automated management and recommendation systems. The classification of musical genres has drawn attention as a very efficient way of organizing digital music libraries [1, 2].

Most of the studies on this type of categorization use the extraction of attributes such as rhythm, melody and timbre as one of their main steps [3–5]. Among these attributes, rhythm plays a very important role in defining the musical style [6]. The study of rhythmics in music signals includes investigating the regularity of its transients, which can be considered as “peak intensity homogeneity” (PIH). Signal PIH can provide relevant information about this feature of rhythmic activity (i.e. it is the result of a set of musical events that constitute the musical information contained in a piece of audio), and thus significantly contributes to classification systems.

The beat histogram has been a widely used tool to help define differences between musical genres by detecting the rhythmic “self-similarity” [7]. Other ways of studying signal self-similarity have also been explored [8, 9].

As an alternative to the techniques used in the signal processing field, this paper proposes studying rhythmic PIH using four properties of audio-associated visibility graphs. We will call the set formed by these four properties the Audio Signal Visibility Descriptor (ASVD).

## Materials and method

Part of the methodology adopted in this article was based on [10].

### Database

The database used in this study was the GTZAN Genre Collection (http://marsyas.info/downloads/datasets.html). This database consists of ten musical genres (Classical, Jazz, Blues, Pop, Rock, Hip-hop, Metal, Disco, Reggae and Country), each with 100 audio files, sample rate 44,100-Hz and 16-bit quantization. This database was proposed by [7] and has been used in many studies involving music information retrieval. The GTZAN database has been established as an important reference in the study of musical genre classification [11].

### Transformation of the {*U*_*i*_} series into a local standard deviation series {*V*_*j*_}

The reduced representation of the audio signal through the local standard deviation series proposed by [8] is calculated as described below. Let {*U*_*i*_} be the series of samples that represents the signal. The total number of points *N* is a function *N* = *S*_*r*_ × *t*, where the sampling rate is reduced to *S*_*r*_ = 11, 000 Hz with a duration of *t* = 30 s. The set {*U*_*i*_} = {*U*_1_, ⋯, *U*_*N*_} is segmented into *m* = 3, 000 nonoverlapping boxes, each with a size of λ = 110 samples. For each box *j* = 1⋯*m* the standard deviation is calculated. In Eqs 1 and 2, the sum is over the elements inside the box of size of λ. At the *j*^*th*^ box, we have:

$$\begin{array}{c} V_{j}=\sqrt{\frac{\sum_{(j-1)\cdot \lambda +1}^{j\lambda }(U_{i}-\bar{U_{j}}{)}^{2}}{\lambda -1}}, \end{array}$$ (1)

where the mean is given by Eq 2.

$$\bar{U_{j}}=\frac{\sum_{(j-1)\cdot \lambda +1}^{j\lambda }\left(U_{i}\right)}{\lambda }$$ (2)

Therefore, a series {*V*_*j*_} = {*V*_1_, *V*_2_, ⋯, *V*_*m*_} with *m* = 3, 000 points is created. In this article, we refer to the series {*V*_*j*_} as a series of local standard deviation.

**Fig 1:**
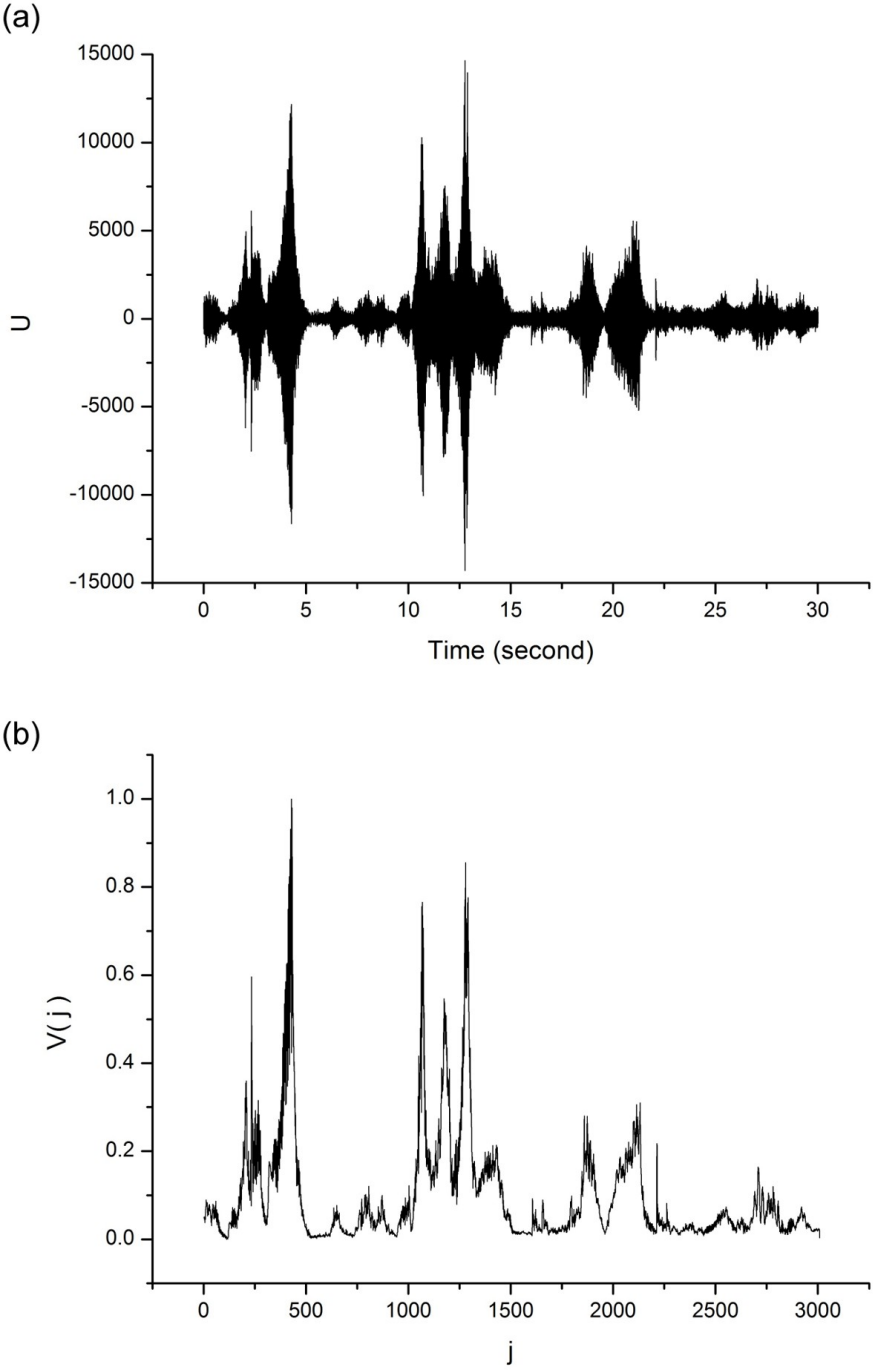
(a) Series corresponding to the 30 s sample of a string quartet by Claude Debussy, (b) local standard deviation series of the signal represented in (a).

Fig 1(a) shows a 30 s audio excerpt from the musical piece *Ainsi La Nuit*, a string quartet by composer Claude-Achille Debussy, along with its respective local standard deviation series {*V*_*j*_}.

### Transformation of series {*V*_*j*_} into a visibility graph

The graph is a mathematical structure *G* = (*V*, *E*), where *V* is the (finite and nonempty) set of vertices and *E* is the set of edges (pairs of unordered vertices).

Each point in the series {*V*_*j*_}, with *j* = 1⋯*m*, is considered a vertex of the graph. We consider each vertex as an ordered pair (*j*, *V*_*j*_), where *j* is the position of the point in the series. Eq 3 defines the criterion used to link two vertices [12]. According to this criterion, two vertices (*a*, *V*_*a*_) and (*b*, *V*_*b*_) are connected if every point (*c*, *V*_*c*_) between *V*_*a*_ and *V*_*b*_ satisfies:

$$\begin{array}{c} \frac{V_{b}-V_{c}}{b-c}>\frac{V_{b}-V_{a}}{b-a} \end{array}$$ (3)

Eq 3 gives the comparison between the slope of the line passing through points (*b*, *V*_*b*_) and (*c*, *V*_*c*_)—*α*_*bc*_—left side of the equation—and the slope of the line passing through (*b*, *V*_*b*_) and (*a*, *V*_*a*_)—*α*_*ba*_—right side of the equation. Every time that *α*_*bc*_ > *α*_*ba*_ for all points (*c*, *V*_*c*_) between (*a*, *V*_*a*_) and (*b*, *V*_*b*_), there is visibility between (*a*, *V*_*a*_) and (*b*, *V*_*b*_), and a link ((*V*_*a*_, *V*_*b*_)) ∈ *E* is created in the graph. Otherwise, no link is created. After applying Eq 3 to all point pairs in the series, we have the local standard deviation series {*V*_*j*_} mapped onto a graph *G*.

**Fig 2:**
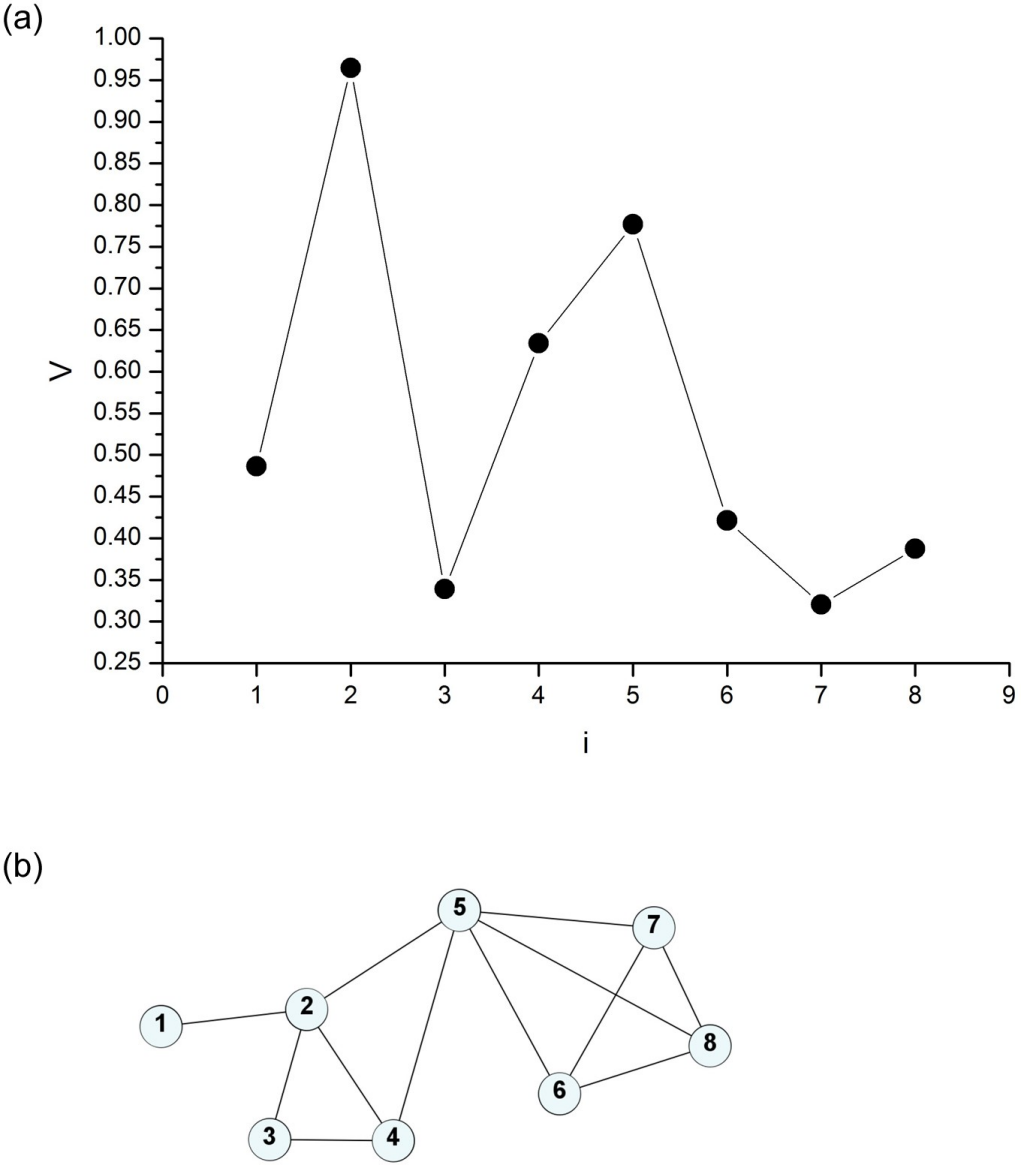
Cartesian representation of a series of eight points {*V*_*j*_} (a) and their respective visibility graph (b).

Fig 2(a) shows an eight-point series in the Cartesian plane and Fig 2(b) shows the visibility graph generated from this series. The numeric values that appear on the label of each vertex correspond to the series {*V*_*j*_}. If we imagine each point in this series as a mountain peak, we can say that every time an observer on one of these peaks can see the other peak in a straight line without being visually blocked by an intermediate peak, a link between their respective vertices is created in the graph [12]. Otherwise, no link between the vertices is created. The higher the signal point is relative to its neighbors, the higher its visibility is, and the more edges it will have in the graph.

**Fig 3:**
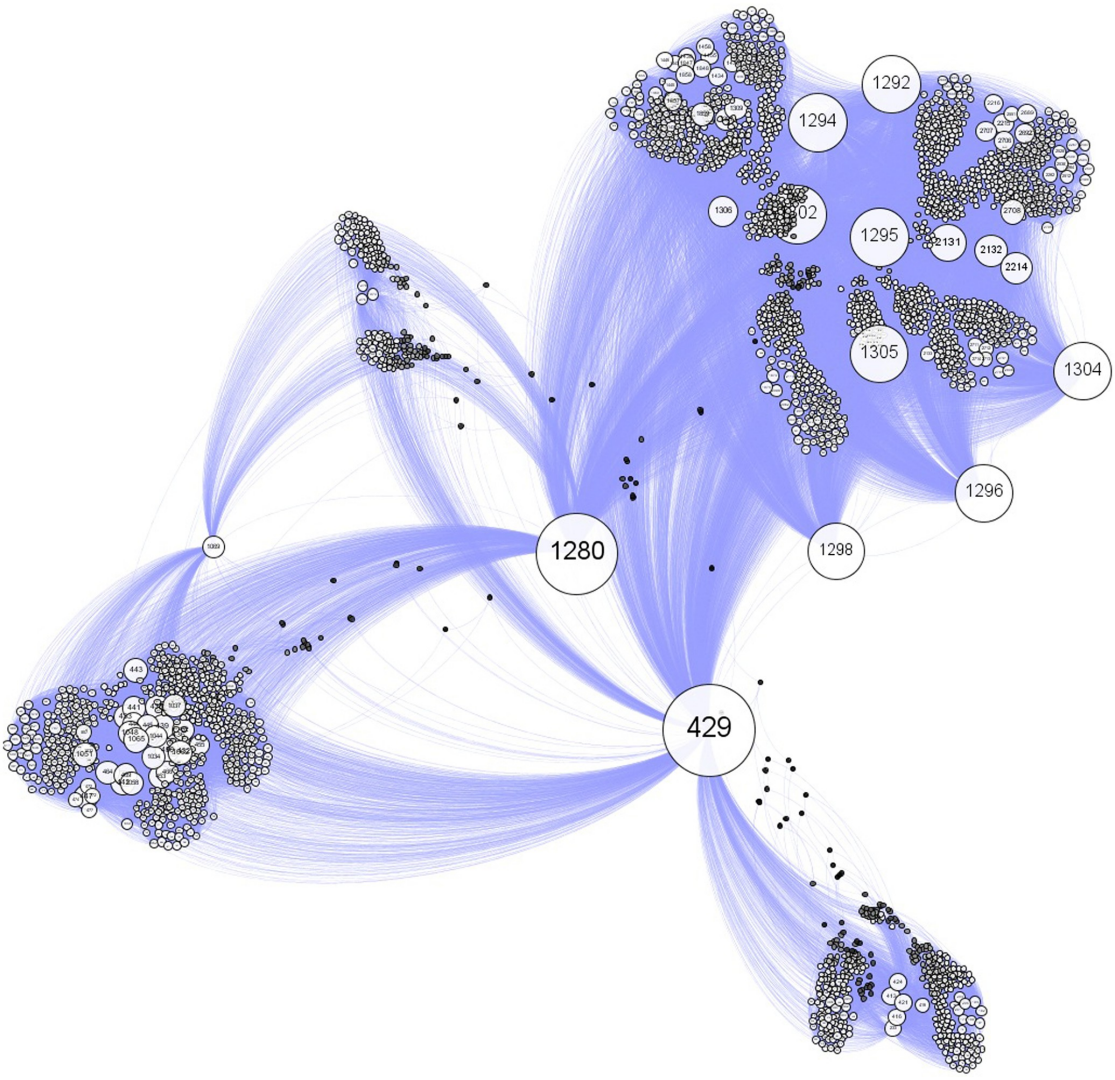
Visibility graph generated from Debussy string quartet {*V*_*j*_} series (Fig 1).

An example of a visibility graph constructed from a musical audio signal is shown in Fig 3. This graph represents the mapping of the series shown in Fig 1(b). It has 3, 000 vertices and 88, 481 edges. The numbers appearing on graph’s vertex labels correspond to the position *j* of each point *V*_*j*_ of the local standard deviation series, and the sizes of the vertices are proportional to their respective degrees. Note that the highest degree vertices correspond to the points in the series that have the highest *V*_*j*_ peaks, such as *j* = 429 (highest degree), *j* = 1280 and *j* = 1292.

### Calculation of the Audio Signal Visibility Descriptor

In this paper, we call the Audio Signal Visibility Descriptor (ASVD) a vector formed by four properties of the visibility graphs [12] mapped from the local standard deviation of the music signals [8]. They are: 1) modularity (*Q*), 2) number of communities (*Nc*), 3) average degree 〈*k*〉 and 4) density (Δ). Each of these properties is considered as an attribute here. The calculation of each of these attributes is presented below.

#### Modularity and number of communities

Modularity is a measure of network structure. This measure is designed to estimate the strength of a division of a network into modules (or communities). A network with a high modularity has dense connections between the vertices within the modules but sparse connections between the vertices in different modules [13]. A high modularity value indicates that the density of edges within communities is higher than expected at random, indicating a good network partition. According to [13], modularity is defined by Eq 4.

$$\begin{array}{c} Q=\frac{1}{2m}\sum_{\left(i,j\right)}(A_{ij}-\frac{k_{i}k_{j}}{2m})\delta \left(c_{i},c_{j}\right) \end{array}$$ (4)

where *i* and *j* are the network’s vertices; *A*_*ij*_ represents the number of edges between *i* and *j*; *k*_*i*_ = ∑_*j*_ *A*_*ij*_; *m* is the sum of all edges of the graph (*m* = |*E*|); and *δ*(*c*_*i*_, *c*_*j*_) is the Kronecker delta function (0 for *c*_*i*_ = *c*_*j*_ and 1 for *c*_*i*_ ≠ *c*_*j*_); where *c*_*i*_ and *c*_*j*_ are the vertex communities.

The maximization of the modularity is computed using the Louvain algorithm [14]:

$$\begin{array}{c} \Delta Q=[\frac{\sum_{in}+k_{i,in}}{2m}-{\left(\frac{\sum_{tot}+k_{i}}{2m}\right)}^{2}]-[\frac{\sum_{in}}{2m}-{\left(\frac{\sum_{tot}}{2m}\right)}^{2}-{\left(\frac{k_{i}}{2m}\right)}^{2}] \end{array}$$ (5)

where ∑_*in*_ is the sum of the edges within community *C*; ∑_*tot*_ is the sum of the incident edges at the vertices in *C*; *k*_*i*_ is the sum of the edges that link *i* to the vertices in *C*; *k*_*i*,*in*_ is the sum of the edges incident on vertex *i*; *m* is the sum of the edges in the network.

#### Average degree

The degree of a vertex corresponds to the total number of its edges. Let *k*_*i*_ be the degree of the *i*^*th*^ vertex of a network. The average degree of a network with *N* vertices is the arithmetic mean of *k*_*i*_.

$$\begin{array}{c} \left\langle k\right\rangle =\frac{1}{N}\times \sum_{i=1}^{N}k_{i} \end{array}$$ (6)

This parameter measures the average connectivity strength of each network vertex. In the visibility graphs, this measure can be interpreted as the average local visibility level of the signal peaks. Signals in which few high local visibility peaks predominate will generate visibility graphs with higher average degrees than signals with many low local visibility peaks. In this sense, the average degree of an audio-associated visibility graph is sensitive to intensity nuances of the music information contained in the audio spectrum. Music signals of styles that use significant percussive activity and intense, regular and persistent rhythmic attacks will be associated with visibility graphs with lower 〈*k*〉 values when compared to the average degrees of graphs generated from music audios that have a greater variety of dynamics nuances and less rhythmic persistence.

#### Density

Let *N* be the number of vertices of a graph. The density Δ is the ratio of the total number of a network’s edges (*m* = |*E*|) to the largest possible number of edges.

$$\begin{array}{c} \Delta =\frac{2\times m}{N(N-1)} \end{array}$$ (7)

The density measures the overall level of network connectivity. In the visibility graphs associated with the audio signals, this measure indicates the overall visibility level of these signals. The higher the level of rhythmic persistence in the signal, the lower the overall visibility, and the lower the density.

### Peak intensity homogeneity of music signals

In this paper, we use peak intensity homogeneity, equivalent to the meaning adopted by [7]. The authors consider that audio recordings with music excerpts that have very strong and persistent beats will produce very PIH signals and that the lower are the persistence and strength of the main beats, the lower the PIH. For the authors, this perception occurs during the calculation of the autocorrelation function, which is during the beat histogram construction process.

**Fig 4:**
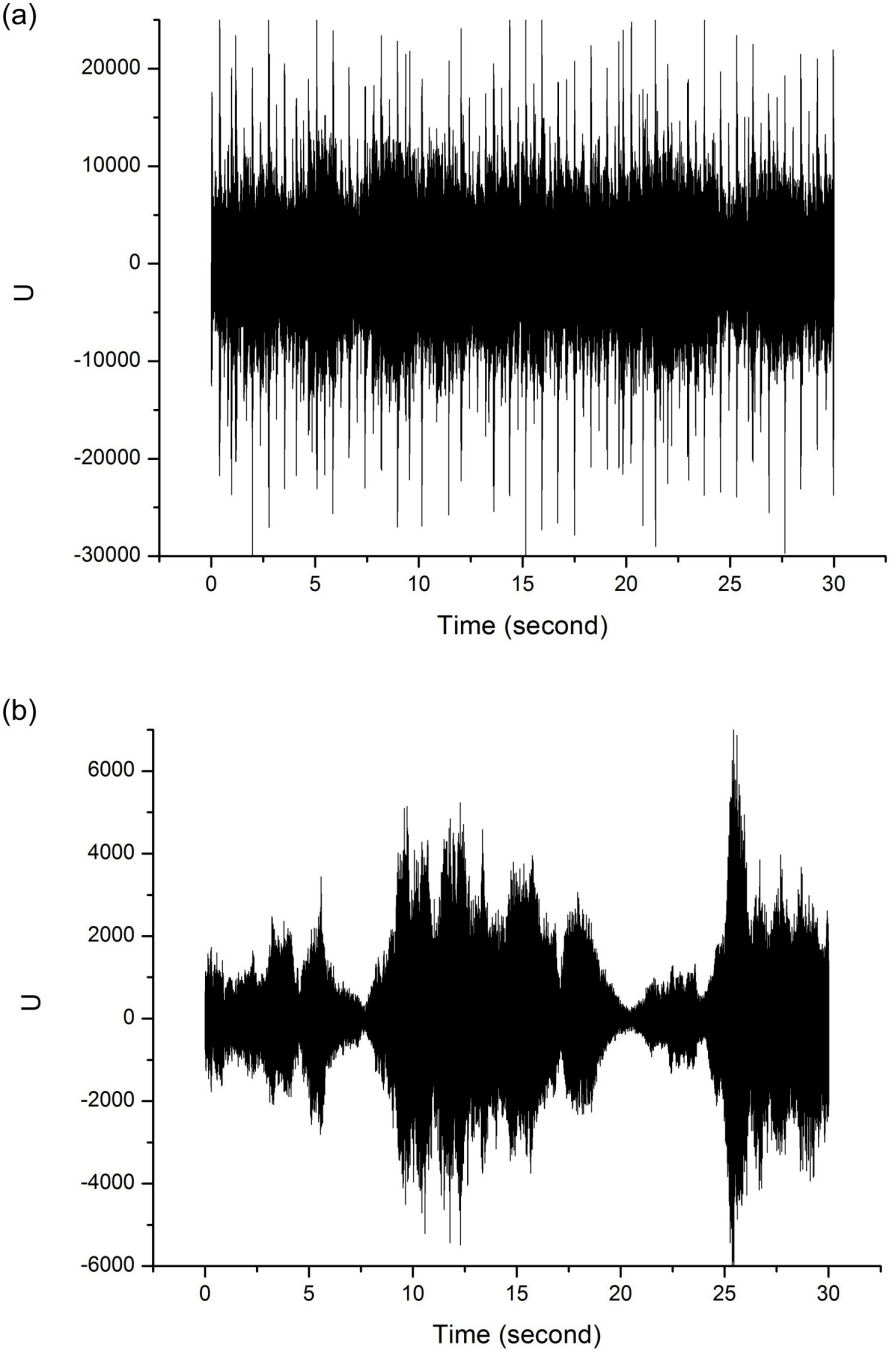
(a) Music signal with strong PIH (heavy metal style); (b) music signal with poor PIH (classical style).

In Fig 4(a) we have a very homogeneous signal, low local visibility and strong PIH, while Fig 4(b) shows a heterogeneous signal with high local visibility and low PIH.

### Audio Signal Processing Descriptors

We use the following descriptors: Mel-Frequency Cepstral Coefficients, Spectral Flux, Zero Crossing Rate, Loudness, Dynamic Complexity, Onset Rate, Detrended Fluctuation Analysis Exponent, Beats Per Minute, and Beat Histogram (First Peak BPM, First Peak Weight, First Peak Spread, Second Peak BPM, Second Peak Weight and Second Peak Spread).

In Appendix section (DETAILS OF AUDIO SIGNAL PROCESSING DESCRIPTORS USED), we will introduce the basics of some of the state-of-the-art descriptors in the audio signal processing field. The computational implementation of these descriptors was performed using the algorithms available in the Essentia library [15].

### Attribute selection

One way of measuring the relative importance of attributes within a classification system is by calculating the information gain from each of these attributes in a decision tree-based structure.

In this study, we use the Ranker + GainRatioAttributeEval algorithm from the machine-learning software WEKA 3.6.9 [16]. It has a tool to identify the most significant attributes in a J48 decision tree, which is a WEKA version of the C4.5 algorithm. At the end, the attribute with the highest gain ratio is selected as the division attribute. The nonterminal vertices of the generated tree are considered as the relevant attributes [17].

## Results

### Standard deviation series calculated from audio signals

**Fig 5:**
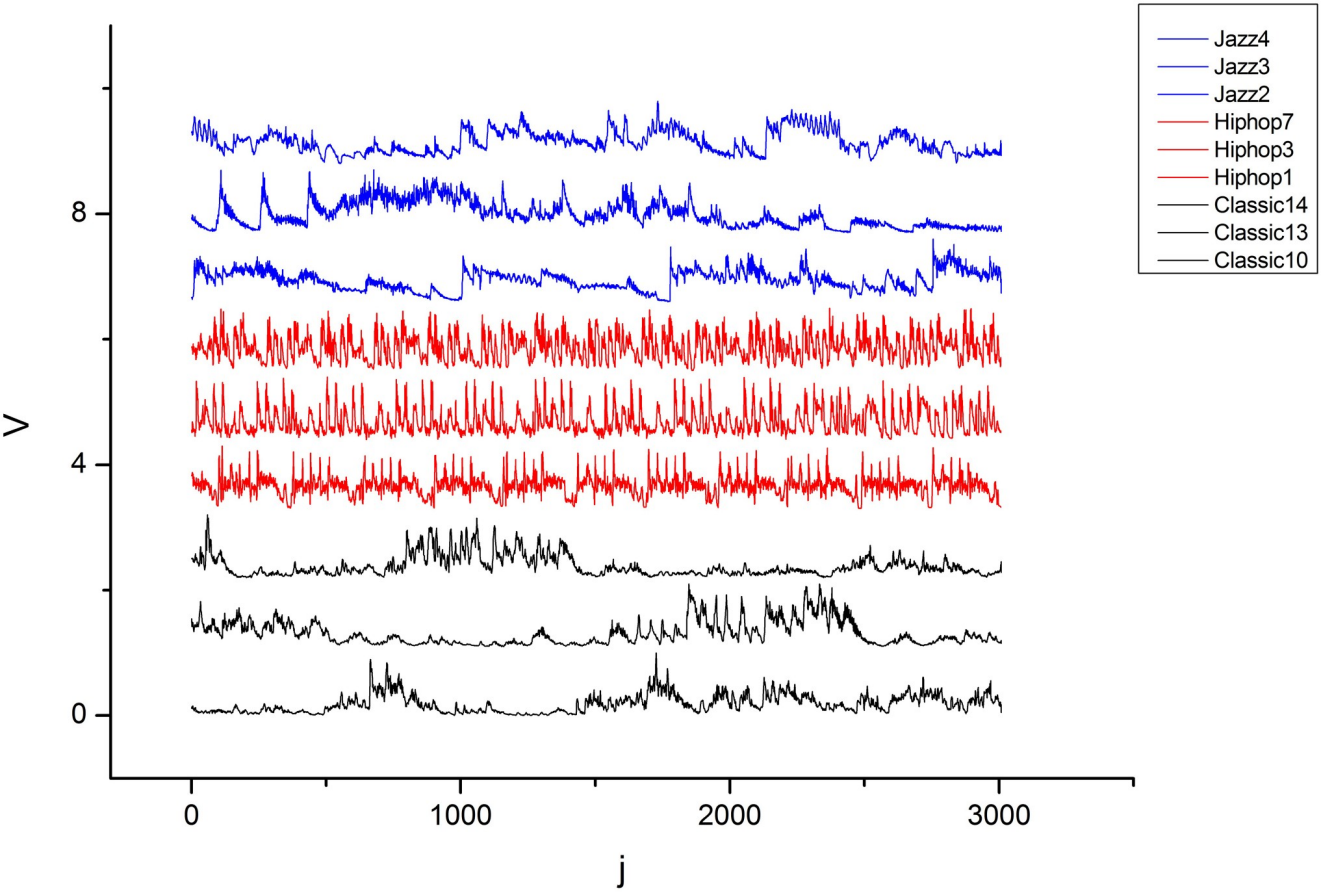
Local standard deviation series of 30 s audio samples. The color blue represents the jazz genre, and red and black represent the hip-hop and classical genres, respectively.

We transformed each of the 1, 000 30 s audio samples into a series of local standard deviation {*V*_*j*_}, representing the original music signals. Fig 5 shows a series of local standard deviation for three musical styles (classical, jazz and hip-hop). If we compare the behavior of the transients within each cluster and between a pair of clusters, different trends are observed between each pair, in addition to a pattern between the signals within each genre.

For example, the hip-hop style (in red) has a greater PIH in its signals when compared to the other two genres. One might initially speculate that there is a PIH hierarchy that would place the classical, jazz, and hip-hop musical genres in ascending order, where the classical style would have the lowest PIH and hip-hop would have the highest PIH among the three, with jazz in an intermediate position (Fig 5). It might also be inferred that these differences are due to the aesthetic choices of each musical style. Thus, the high rhythmic persistence found in hip-hop, due to the rhythmic and instrumental choices inherent to the style, would be more moderate and less persistent in jazz, and even less so in the classical style, where there are typically greater variations in the dynamics and rhythmic nuances.

At this point, it is not yet clear whether these trends will be preserved for the entire database, given that within the same style, one can find signals with completely different patterns or with features similar to signals of another style. Signals with the same PIH features of hip-hop or jazz can be found in the classical style, and consequently with numerical coefficients that indicate patterns that place them in the same cluster. It should not be overlooked that this type of situation is inherent to the field of classification through pattern recognition for both quantitative and qualitative approaches. The observations made from Fig 5 bring an initial reflection on aspects that will be addressed during the presentation of the results in the following sections.

### Audio-associated visibility graphs

**Fig 6:**
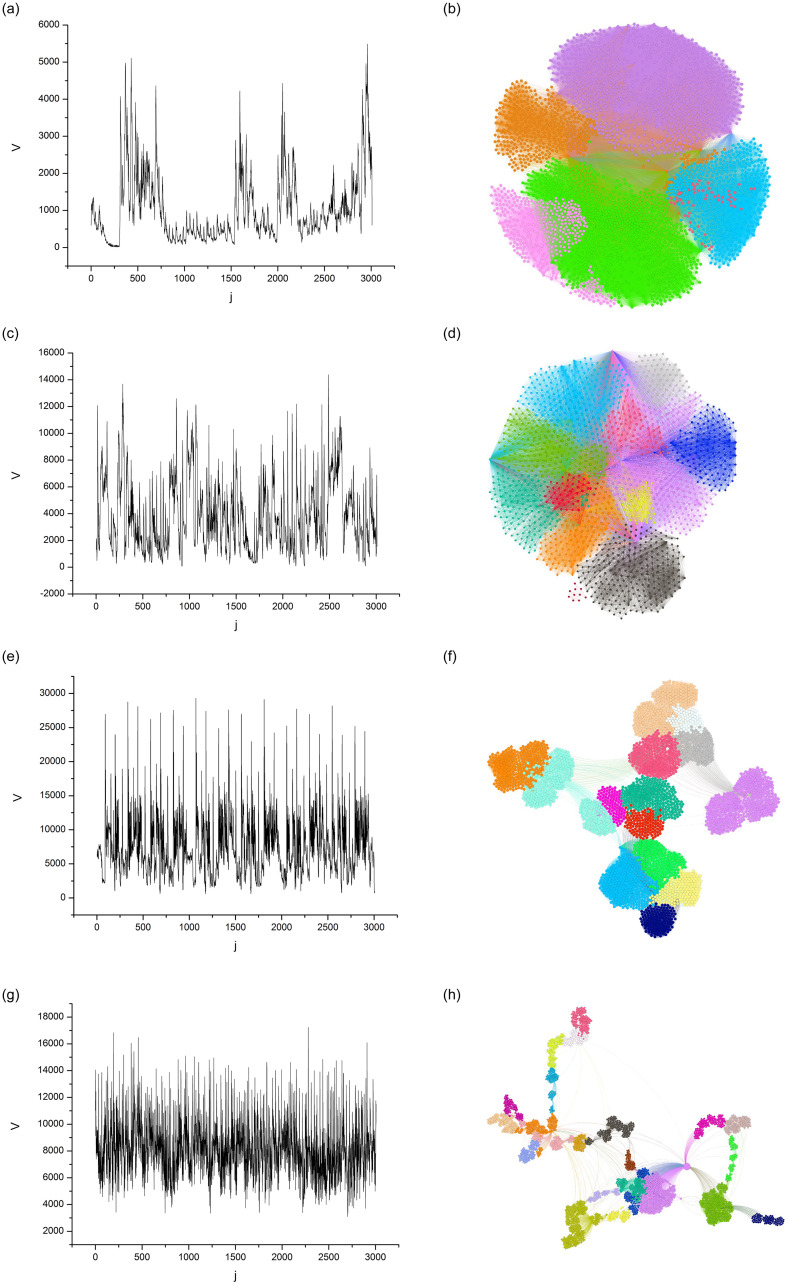
{*V*_*j*_} Series (left) and their respective visibility graphs (right). The colors in the graphs are the communities, which are obtained from modularity.

For each local standard deviation series, we generated a visibility graph, i.e., we built a total of 1, 000 graphs. Fig 6 shows four {*V*_*j*_} series of distinct musical genres and their respective visibility graphs. The different colors in the graphs are communities identified through the modularity algorithm [14], the computational modeling of which was performed in Gephi 0.9.0 (available at https:gephi.org). For all graphs, we used the random mode, which is an option given by the software to produce better decomposition, and the default resolution of 1.0 (more details in [18]). We also used the same visualization algorithm and vertex distribution options.

The algorithm termination criterion was the best visualization of the clusters. In the final result, we observe that graphs with a larger number of communities (or modularity classes) have graphs with smaller and more scattered nodes for easier visualization. In Fig 6, we can observe a correspondence between the persistence features of signal transients and the topological features of community detection in their associated graphs. We can also note that as PIH increases, the modularity and number of communities also increase, while the average degree and density decrease. This feature suggests that the visibility descriptor parameters can be used to hierarchize a set of signals according to the PIH of their transients.

### Hierarchy according to peak intensity homogeneity

Table 1 shows the mean and standard deviation of the modularity, the number of communities, the average degree and the density of the visibility graphs corresponding to 100 audio samples grouped into 10 musical genres.

**Table 1:** Mean and standard deviation of the topological properties of visibility graphs.

	$\bar{Q}$	*σ*_*Q*_	$\bar{Nc}$	*σ*_*Nc*_	$\bar{\langle k\rangle }$	*σ*_〈*k*〉_	$\bar{\Delta }\left(\%\right)$	*σ*_Δ_
**Classical**	0.592	0.120	9.57	3.24	41.71	12.89	1.41	0.48
**Jazz**	0.701	0.083	12.25	3.40	29.04	9.12	0.97	0.37
**Blues**	0.794	0.068	21.23	3.36	22.60	9.93	0.75	0.34
**Reggae**	0.784	0.096	13.95	2.75	21.24	5.21	0.70	0.21
**Pop**	0.882	0.041	15.71	4.75	20.83	5.54	0.69	0.21
**Country**	0.850	0.056	18.75	3.57	20.18	6.94	0.68	0.26
**Hiphop**	0.809	0.077	14.61	2.85	17.75	3.93	0.59	0.16
**Rock**	0.743	0.085	12.49	3.13	16.86	4.33	0.56	0.17
**Disco**	0.854	0.052	19.29	2.85	16.48	4.5	0.55	0.17
**Metal**	0.815	0.073	16.31	3.39	12.3	2.7	0.44	0.12

*Q* (modularity), *Nc* (number of communities), 〈*k*〉 (average degree), and Δ (density).

**Fig 7:**
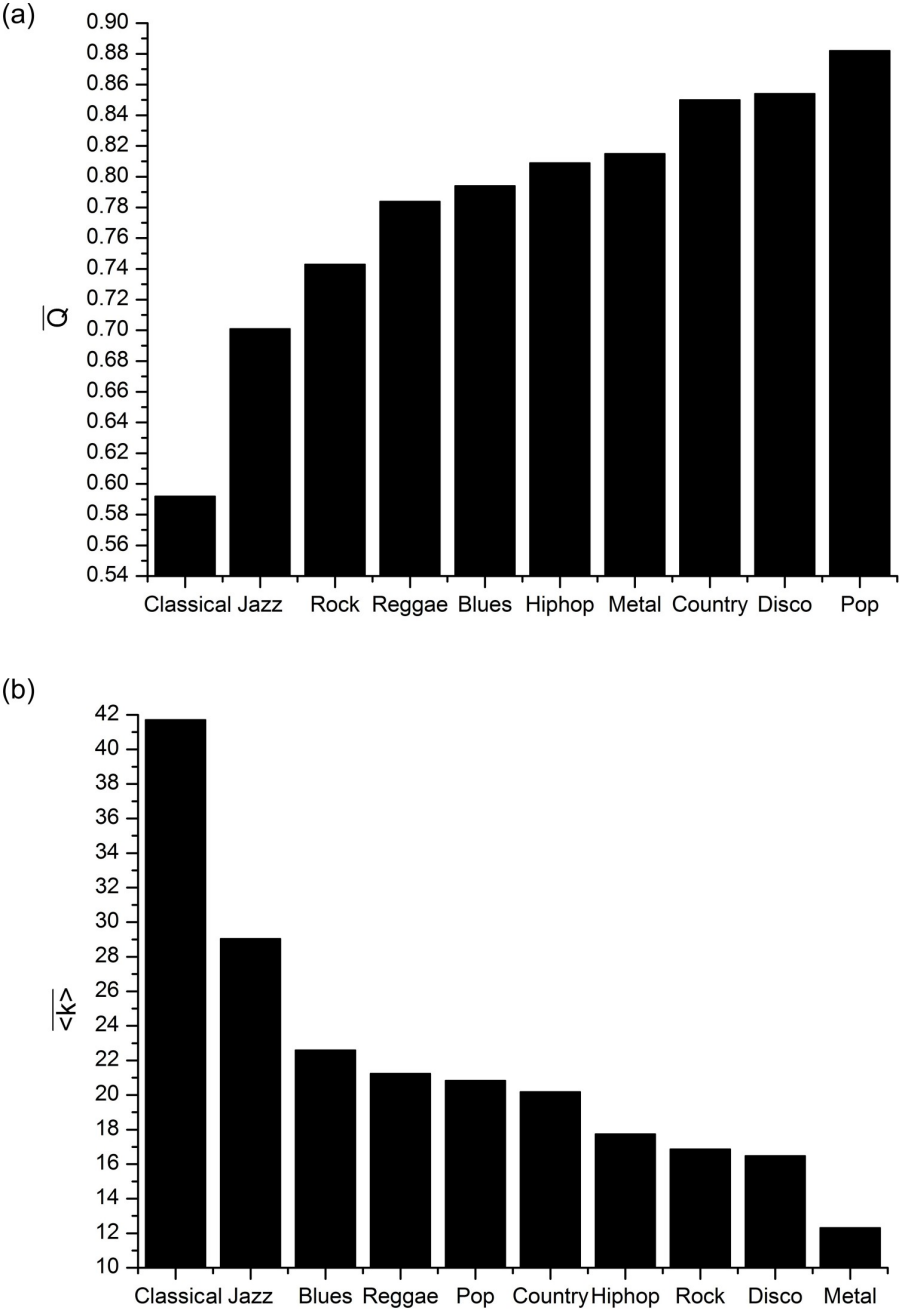
Mean *Q* (a) and 〈*k*〉 (b) calculated from 100 visibility graphs labeled in 10 musical genres.

If we rank the means of the average degree ($\bar{\langle k\rangle }$) and density ($\bar{\Delta }$) in descending order, we will obtain the same sequence of musical genres. Among the ascending orders of $\bar{Q}$ and $\bar{Nc}$, there was a difference in the position for only three clusters (Blues, Hip-hop and Pop). For the four ASVD components, the mean values for Classical, Jazz, Rock, Reggae and Pop were preserved in the same position when placed in descending order for $\bar{\langle k\rangle }$ and $\bar{\Delta }$, and in ascending order for $\bar{Q}$ and $\bar{Nc}$. If we use $\bar{Q}$ and $\bar{\langle k\rangle }$, in that order, to think of a hierarchy, we can consider Fig 7(a) and 7(b) as representations of musical genres in ascending order of PIH. Thus, the musical genre that has the least self-similar signals is Classical, as noted by the large difference in the $\bar{Q}$ and $\bar{\langle k\rangle }$ values of the Classical genre relative to all other genres. The genres whose signals have the highest PIH are Metal, Disco, and Hip-hop.

Jazz is the genre closest to Classical but there are considerable differences between them. For all four components, Reggae occupies the intermediate position. This type of hierarchical organization corroborates the idea that musical genres that opt for very “dense”, “intense”, and “persistent” instrumental arrangements have signals with higher PIH and tend to occupy positions opposite to genres with instrumental textures richer in dynamics, and therefore with less PIH in their signals. In an intermediate position are musical styles that seek to balance the aesthetic influences of both extremes. In many cases there may be no significant differences between the clusters established by *Q*, 〈*k*〉, Δ, and *Nc*. To study this aspect, we performed a hypothesis test for pairwise comparisons between the mean $\bar{Q}$, $\bar{\langle k\rangle }$, $\bar{\Delta }$, and $\bar{Nc}$.

**Fig 8:**
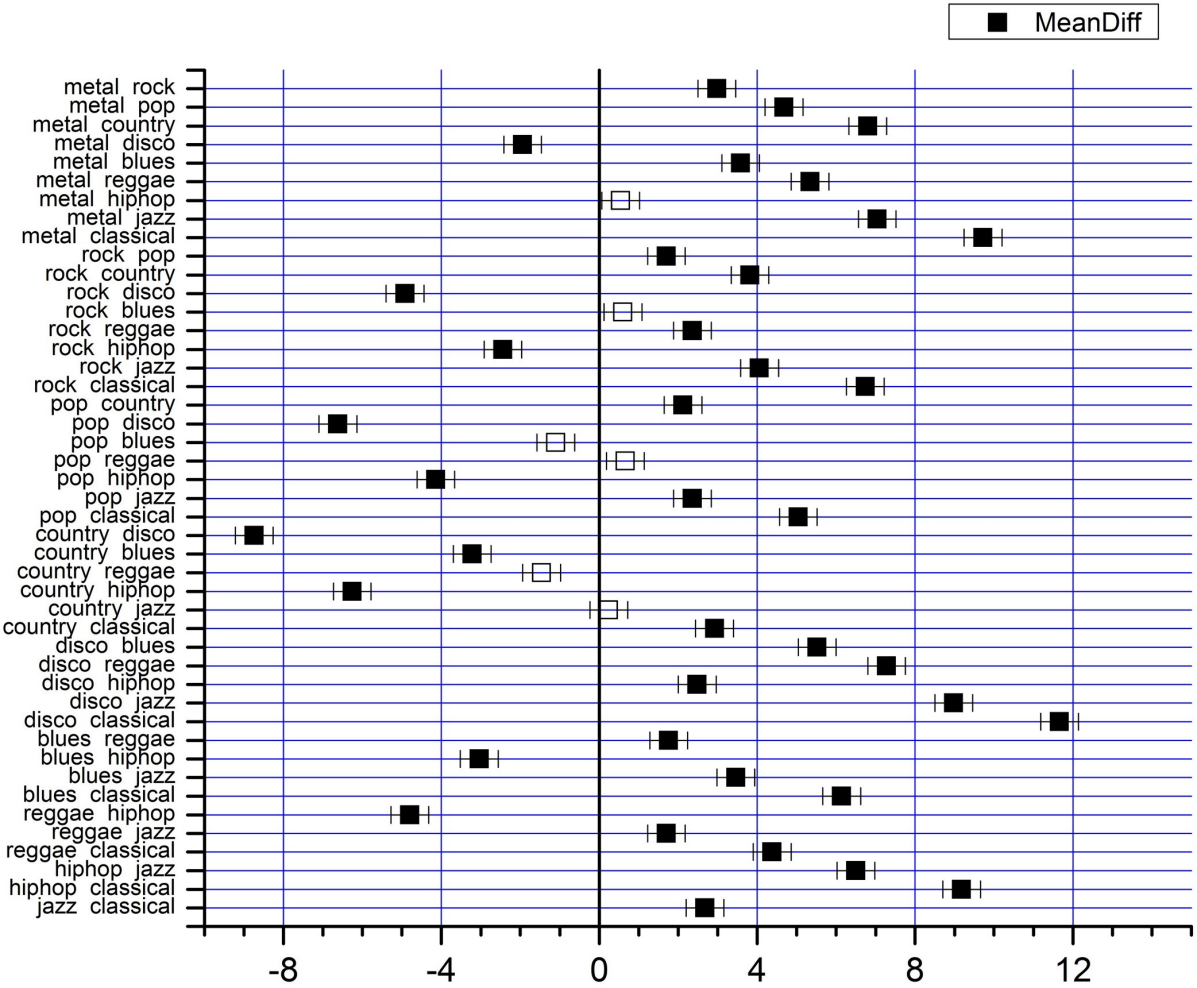
Difference in the mean *Nc* between pairs of musical genres according to Tukey’s test. Black boxes represent statistically significant differences, and white boxes represent nonsignificant differences.

Table 2 shows the percentage of pairs of musical genres that have significant differences according to Tukey’s test, adopting a confidence interval of 0.95. We note that the means of the four components of the ASVD vector respond positively to the hypothesis of significant differences for most pairs of musical genres. For example, 39 of the 45 genre pairs hypothesized to have a significant difference for $\bar{Nc}$ (mean number of communities) showed a positive response (Fig 8). Specifically, between the Classical style and all the others, the test was positive. Conversely, Metal and Hip-hop, Rock and Blues, and Country and Jazz were the only pairs with rejected hypothesis. These results vary slightly for $\bar{Q}$, $\bar{\langle k\rangle }$ and $\bar{\Delta }$, but retain the same overall characteristic. This shows that $\bar{Q}$, $\bar{\langle k\rangle }$ and $\bar{\Delta }$ can be used as representative parameters for most musical genres used in this experiment. Although the rejection of the hypothesis of significant differences between some pairs suggests that they may be labeled within the same cluster, we note that the ASVD components have detected an overall PIH trend for the musical genres in this database that follows an increasing order for $\bar{Q}$ and $\bar{Nc}$ and a decreasing order for $\bar{\langle k\rangle }$ and $\bar{\Delta }$, which can be interpreted according to the intuitive notion suggested by the label of each genre. This trend reinforces the hierarchy presented in Fig 7.

**Table 2:** Pairs of musical genres with significant differences for clusters formed with the ASVD components, according to Tukey’s test.

ASVD	Pair of musical genres with significant difference (%)
Nc	86.7
Q	77.8
〈*k*〉	75.6
Δ	71.1

Until this point, the techniques necessary to distinguish between clusters have not been used. So far, the evidence of trends that may be useful in classifying musical genres has been detected.

### Machine learning and classification

Machine learning and classification were performed with supervised artificial neural networks taking two scenarios into consideration. In the first, we used an attribute vector with only the ASVD (modularity, number of communities, density and average degree). The idea was to explore a situation where only this descriptor of rhythmic activity was used. Next, we performed learning and classification with only the Beat Histogram (BH). Then, we compared the results.

In the second scenario, which also used neural networks, experiments were performed using each of the descriptors in the first scenario separately, adding the timbre, dynamics and onset detection descriptors. First, we set up an attribute vector by joining the ASVD with 18 ASPD (Audio Signal Processing Descriptors: 13 MFCCs, Spectral Flux, Zero Crossing Rate, Onset Rate, Loudness and Dynamic Complexity). Next, we performed machine learning by combining the BH to the same ASPD. Then, we compared the results again. In Appendix section (SCHEMATIC EXPLANATION OF THE ARTIFICIAL NEURAL NETWORKS), we present some details of the artificial neural networks used.

#### Scenario 1

The neural network that presented the best results for the first scenario was a network with a hidden layer of 16 neurons. On average, 39% of instances were correctly classified. The confusion matrix in Table 3 shows the results of the classification with artificial neural networks using only the four ASVD parameters as input attributes. In the main diagonal, where we have the true positives, we observe that the Classical, Disco, Metal and Pop genres had the highest rate of true positives (68, 67, 55 and 55%, respectively). This indicates that in these cases, the PIH patterns described by the ASVD were better interpreted by the neural networks than the other musical genres. We can speculate that these genres have more typical features according to the classification system used. The lowest hit rate was found for Blues. This indicates that, in the vast majority of cases, the PIH pattern of its signals is not typical enough to strongly characterize its cluster in this particular system. This situation was expected because the use of a single attribute type in the classification is not sufficient to achieve a high hit rate for all clusters, as it is natural for audio signals to share PIH characteristics.

**Table 3:** Classification using artificial neural networks and ASVD attributes.

	Classified as									
	**Blues**	**Clas**	**Coun**	**Disco**	**Hip**	**Jazz**	**Metal**	**Pop**	**Regg**	**Rock**
**Blues**	**6**	4	5	13	2	14	15	5	14	12
**Class**	1	**68**	7	0	0	18	0	1	4	1
**Coun**	3	6	**47**	0	2	9	6	0	14	13
**Disco**	0	1	0	**67**	9	0	6	3	4	10
**Hip**	3	1	2	31	**11**	1	7	19	11	14
**Jazz**	4	22	15	1	1	**37**	3	4	10	5
**Metal**	0	0	5	21	2	0	**55**	1	9	4
**Pop**	1	0	0	4	0	0	8	**55**	1	15
**Regg**	1	5	10	3	4	6	1	15	**48**	7
**Rock**	1	0	15	13	3	6	19	3	16	**24**

**Fig 9:**
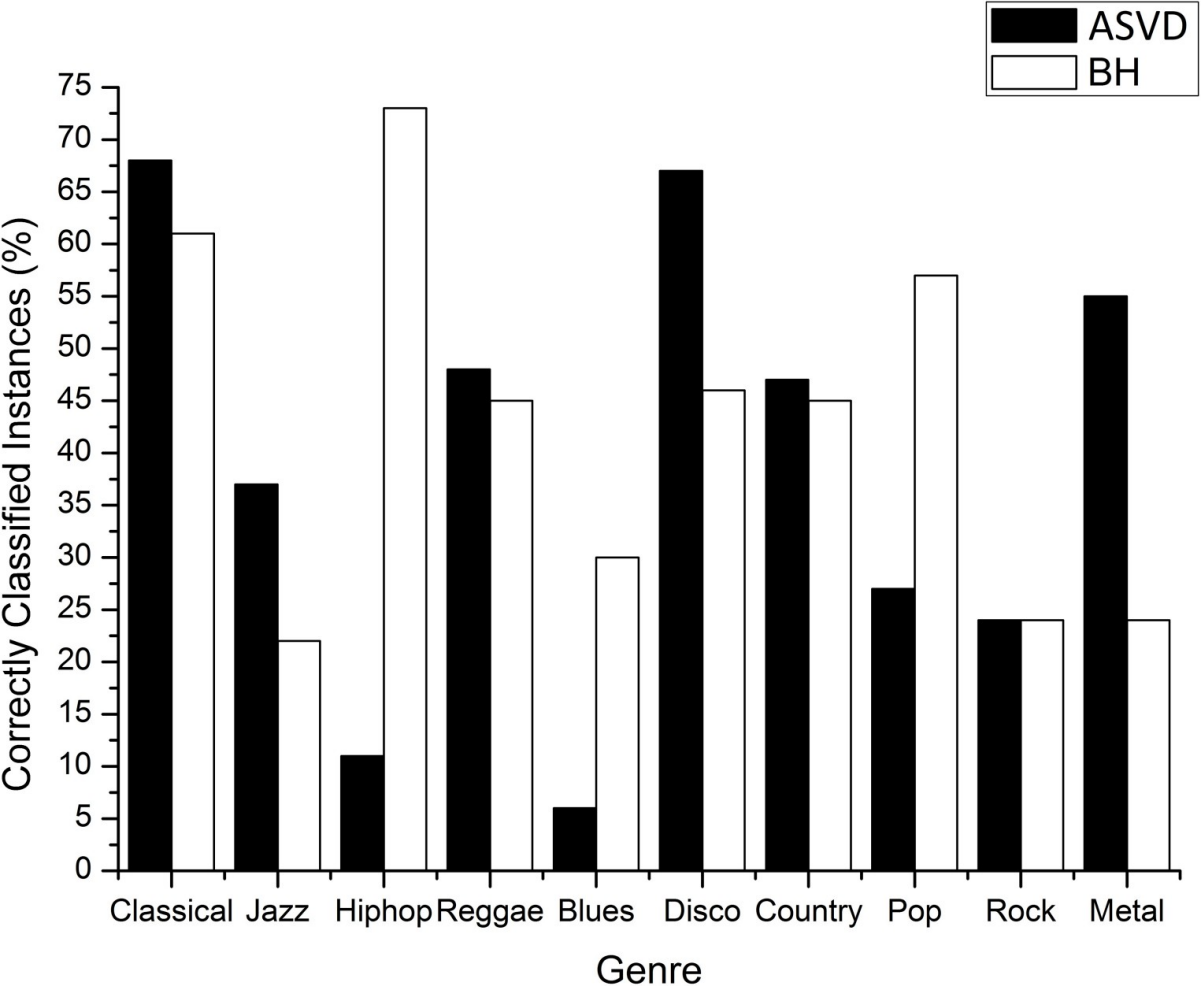
True positives rate for classification using neural networks, where the input attributes are the ASVD or beat histogram.

Using only the six beat histogram parameters as input attributes in a neural network with a hidden layer of 16 neurons, we obtained a mean true positive rate of 48.3%. Although the mean value is higher than the value obtained with the ASVD, when we compare the rate of instances correctly classified by musical genre using just the ASVD and that using just the beat histogram, we found that the ASVD achieves, for seven of the ten genres, hit rates that are higher than or equal to those achieved by the classification using the beat histogram (Fig 9). The ASVD classification outperforms the beat histogram for the Classical, Jazz, Reggae, Disco, Country and Metal genres and ties for the Rock genre.

#### Scenario 2

For the second scenario, where in addition to the rhythm descriptors, descriptors of other aspects of the signal were used, the best result was obtained by a neural network with two hidden layers, each with 32 neurons, obtaining a result of 76.7%. In this case, the input set (*x*_*i*_) was formed by 22 attributes extracted from an audio signal, four being the ASVD and 18 Audio Signal Processing Descriptors (ASPD). The output *y*_*k*_ was the music class attributed to the respective signal based on the pattern recognition provided by the descriptors.

**Fig 10:**
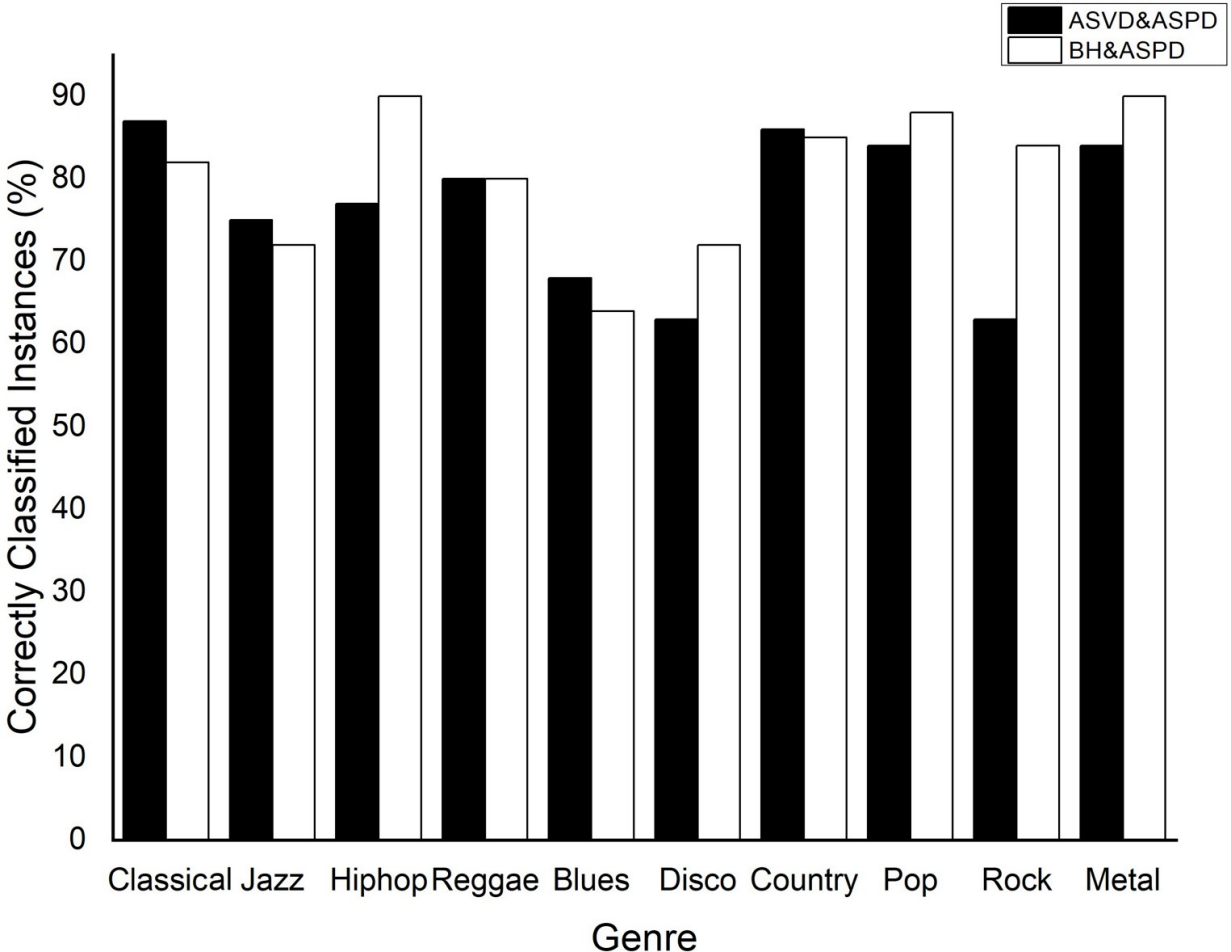
Results of classification with neural network: Audio Signal Visibility Descriptor (ASVD) & Audio Signal Processing Descriptors (ASPD) compared with beat histogram & ASPD.

When we built the neural network using a hidden layer of 32 neurons and 24 input attributes (six beat histogram parameters plus the same 18 ASPD attributes), we obtained an average accuracy of 80.7%. When comparing the classification performance per musical genre of the input configuration using the ASVD + ASPD and BH + ASPD, despite a mean accuracy of 76.7%, looking at each genre individually case by case, we note that in half of the musical genres the percentage of true positives with the ASVD + ASPD exceeded or equaled the results using the beat histogram (Fig 10). This indicates that the use of the ASVD as a descriptor of rhythmic activity in a classification system is comparable to the beat histogram used in the same function in a similar system.

### Gain ratio ranking

**Fig 11:**
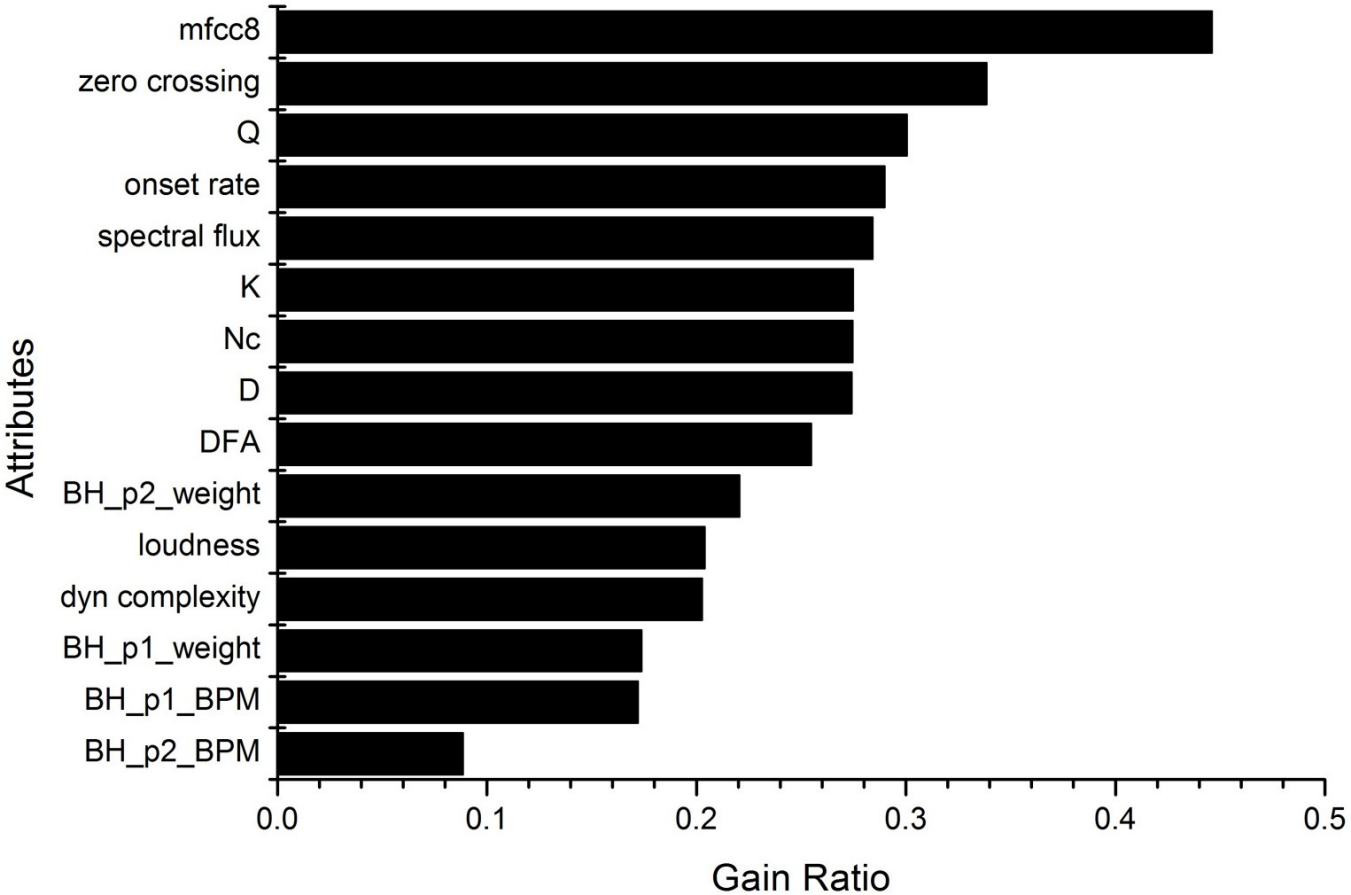
Gain ratio for the attribute selection.

To select the attributes, we used the *Ranker* + *GainRatioAttributeEval* algorithm in WEKA 3.6.9. The attributes extracted for selection were: the ASVD (modularity—*Q*, number of communities—*Nc*, average degree—〈*k*〉, density—Δ), 13 MFCCs, Beat Histogram (bh-p1, bh-p1-spread, bh-p1-weight, bh-p2, bh-p2-spread, bh-p2-weight), Loudness, DFA Exponent, Dynamics Complexity, Spectral Flow, Onset Rate, and Zero Crossing Rate. Fig 11 shows the ranking of the best gain ratios among these attributes. Among the 13 MFCCs, we highlight only MFCC 8, which achieved the best information gain ratio. The other 12 MFCCs occupied positions in all ranges and were omitted to simplify the analysis and to focus on other attribute types.

The results show that the two best gain ratios were obtained with two timbre descriptors (MFCC 8 and Zero crossing rate). In third place was *Q* as the visibility descriptor representative, which was higher than the six beat histogram attributes and the traditional audio signal processing field descriptors such as Spectral Flow, Onset Rate and Loudness. The other visibility descriptors (〈*k*〉, *Nc* and Δ) occupy the sixth, seventh, and eighth positions, respectively, all reaching similar ratios and ranking ahead of the DFA Exponent self-similarity descriptor and the three other time-frequency descriptors. Overall, the visibility descriptors occupied a good position in relation to the timbre descriptors, and an excellent position in relation to the rhythm descriptors.

## Comparison with related studies

The 76.7% mean accuracy using the ASVD + ASPD attributes exceeded those found in some studies with the same database and similar classification systems, for example, [7] (61%), [19] (74%), [20] (74.5%), and [21] (58.07%), and is comparable to the works of [22] (78.5%), [23] (76.8%), and [24] (78.2%), which also used the same database. To better understand the value of the results found in the classification using the Audio Signal Visibility Descriptor, it is important to note that the mentioned studies have a larger number of attributes, reaching up to 80 [25], compared with only the 22 attributes used in our experiment.

**Fig 12:**
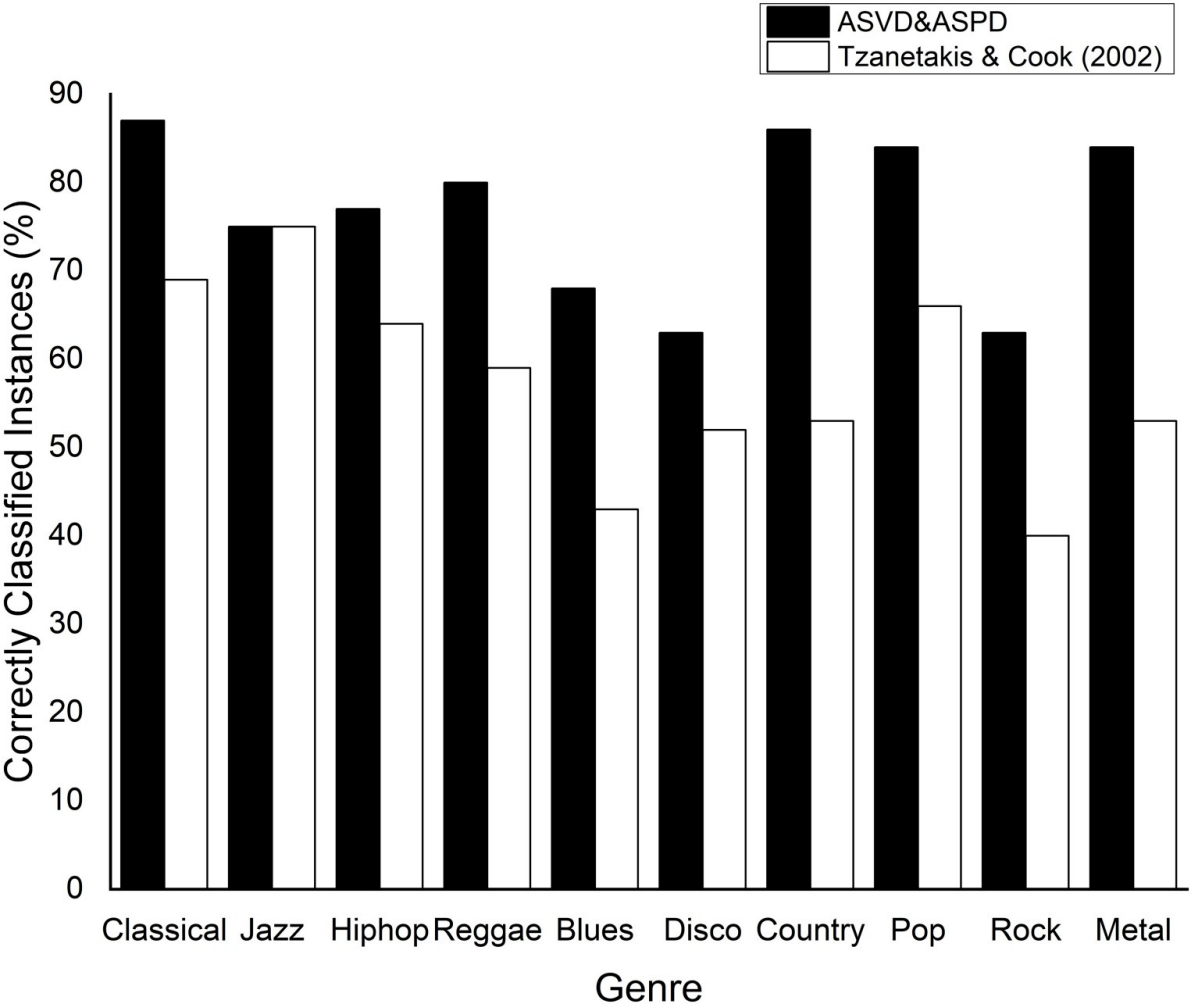
Result of the classification of musical genres in the GTZAN database in our proposal and in the experiment of [7].

The accuracy of the classification of this experiment (black bars) and of the experiment of [7] (white bars) are shown in Fig 12. It can be observed that our work obtained a higher hit rate for all categories except for the Jazz genre. For the seven classes there was a difference ≥19%. For Country and Metal, in particular, there was a noticeable difference in the number of true positives: 32% and 37%, respectively. These results show that, in this case, the classification of musical genres using the ASVD for rhythmic activity extraction instead of the beat histogram resulted in a categorization system with a better overall and individual hit rate. This comparison is very important because the work in [7] has been used as a benchmark in many music information retrieval studies, and this shows how the new feature extraction method used in this work can succeed over a method traditionally used in the literature.

## Conclusion

In this study, we introduced the Audio Signal Visibility Descriptor (ASVD) as a new way to extract features in audio signals for the classification of musical genres using network properties rather than Fourier transform-based algorithms.

We showed that the visibility graphs constructed from audio signals revealed, through graphical representation (Fig 6) of the ASVD parameters (Tables 1 and 2), distinct PIH features associated with the rhythmic activity of musical styles. Based on these results, we proposed a hierarchy according to PIH (Fig 7) and then showed that the modularity, number of communities, average degree and density can be used in classification systems as parameters of the feature’s vector in different scenarios.

In a classification system using only the ASVD, we achieved an average accuracy of 39%. We compared the instances correctly classified by this system with another system using only the beat histogram, and then, in a pairwise comparison of genres, obtained an accuracy higher than or equal to the second system in 70% of the musical genre pairs (Fig 9).

Considering a scenario with 18 audio signal processing descriptors plus the ASVD, the mean accuracy of the classification was 76.7%, which is comparable to or higher than several related studies (Fig 12). In yet another classification experiment using the same 18 attributes as the previous experiment, and using the beat histogram instead of the ASVD, we obtained equal or higher accuracy in half of the ten groups of musical genres (Fig 10).

In studying the attribute selection, we obtained the ASVD parameters among the top positions and at intermediate positions (Fig 11). This outcome shows that the ASVD plays a relevant role in the decision making of the algorithm, as it occupies positions alongside the best attributes for classification. We conclude that the proposed descriptor for this database and for this classification system displayed the ability to identify patterns of rhythmic activity that contributed significantly to the study of the representation, hierarchization and categorization of musical genres.

Based on the results obtained in this experiment, the Audio Signal Visibility Descriptor (ASVD) can be considered as a new alternative for the extraction of features for the retrieval of music information in audio signals and can be successfully used together with the descriptors based on Fourier transforms. In future works, we intend to extend the ASVD vector, adding other metrics used in complex networks.

## Appendix

### Details of Audio Signal Processing Descriptors used

Now we will introduce the basics of some of the state-of-the-art descriptors in the audio signal processing field: Mel-Frequency Cepstral Coefficients, Spectral Flux, Zero Crossing Rate, Loudness, Dynamic Complexity, Onset Rate, Detrended Fluctuation Analysis Exponent, Beats Per Minute and Beat Histogram.

The computational implementation of these descriptors was performed using the algorithms available in the Essentia library [15].

#### Mel-Frequency Cepstral Coefficients—MFCC

These features were first explored in voice processing [26], and then began to have applications in music signal processing and gained much importance in genre classification [7]. These coefficients are a representation of the spectral envelope that is based on the Short-Time Fourier Transform (STFT). MFCCs use a spectral envelope representation that seeks to approximate nonlinear human pitch perception through the also nonlinear Mel scale.

#### Spectral flux

Spectral flux is the quantitative measure of changes in the power spectrum of the signal. The spectral flux is given by the difference between two consecutive STFT frames of the magnitude spectrum, and it plays an important role in the detection of onsets. [27] presents different ways to calculate the spectral flux so it can play the role of a novelty function within an audio signal detection scheme.

#### Zero crossing rate

When successive signals assume values with opposite signs (positive-negative or negative-positive), we say that at this point there was a zero crossing. The zero crossing rate is the ratio between the number of consecutive sign changes and the total number of values.

In the context of studying music signals, the zero crossing rate has been used in musical genre classification systems [28, 29] and in the study of percussive sound separation [30].

#### Loudness

Loudness is defined as an entity related to the perception of sound, while intensity is an entity related to the physical features of sound, whose magnitude can be measured numerically. Therefore, loudness essentially has a subjective nature.

Stevens [31] proposed a way of quantifying the loudness by establishing a relation between the sensation of sound perception and the intensity of the sound (Eq 8).

$$\begin{array}{c} \psi \left(I\right)=kI^{\alpha } \end{array}$$ (8)

*ψ*(*I*) is the magnitude of the subjective sensation given by the sound stimulus; *I* is the magnitude of the physical stimulus, *α* is the exponent for the stimulus given by a sound pressure of 3, 000 Hz tone. For the loudness *α* = 0.67; *k* is a proportionality constant that depends on the units used.

#### Dynamic complexity

Dynamic complexity is calculated by the absolute mean deviation from the overall estimated loudness level on the dB scale. This index reflects the amount of loudness fluctuation in the dynamic range of an audio track.

#### Onset rate

Calculates the number of onsets per second in an audio excerpt. The onset rate is based on a high frequency content method known as the High Frequency Content (HFC) function. According to [32], HFC is more successful at detecting percussive onsets than nonpercussive onsets, such as strings and flute.

#### Detrended fluctuation analysis exponent

The Detrended Fluctuation Analysis (DFA), proposed by [33], is a statistical method that detects long-range correlations present in a nonstationary time series in different scales and measures the level of self-similarity in this time series. According to some authors [34–36], the DFA method has an advantage of allowing the “long-range power-law correlations in signals with embedded polynomial trends that can mask the true correlations in the fluctuations of a noise signal”.

An adaptation of DFA for the study of music audio signals—The Detrended Fluctuation Analysis Exponent (DFA exponent)—was proposed by [8], where a coefficient for calculating the power-law deviations at time intervals of various sizes is presented. With this coefficient, genres such as Dance are associated with low DFA exponent values as a consequence of a low long-range correlation in the time series of its audio signal. In turn, genres such as Hindustani and Classical have high DFA exponent values, reflecting the high long-range correlations in their series. The DFA exponent is also known as danceability [37].

#### Beats Per Mimute—BPM

It is the average of the most salient BPM values that represent periodicities in the signal (the average BPM). The set of features to represent the structure of the rhythm is based on the detection of the most salient signal periodicities. The signal is first decomposed into an octave band frequency series using the discrete wavelet transform (DWT). After this decomposition, each band’s time-domain amplitude envelope is separately extracted. This is achieved by applying full-wave rectification, low-pass filtering and downsampling to each octave frequency band. After the average removal, the envelopes of each band are summed, and the autocorrelation of the resulting sum envelope is calculated. The dominant peaks of the autocorrelation function correspond to the various periodicities of the signal envelope. These peaks are accumulated throughout the sound file into a beat histogram, where each box corresponds to the peak interval, i.e., the beat period in beats per minute (bpm) [7].

The time *t* in BPM of a constant-time music audio segment can be calculated using the time interval Δ*t* in seconds and the number of beats *B* in this segment [27].

$$\begin{array}{c} BPM=\frac{B\times 60}{\Delta t} \end{array}$$ (9)

#### Beat histogram

Automatic beat detection systems in audio signals provide an estimation of the execution and strength of their main rhythm. In addition to these resources for characterizing musical genres, the following information can be used in the attribute vectors: i) regularity of the rhythm, ii) relation of the main beat to sub beats, and iii) relative strength of the sub beats to the main rhythm.

The beat histogram is widely used to detect these additional features. This histogram is constructed as follows. First, using a discrete wavelet transform (DWT) [38], the audio signal is decomposed into a series of octave frequency bands. Subsequently, the amplitude envelope of each band in the time domain is extracted using full-wave rectification, low-pass filtering, downsampling, and mean removal. Next, all the envelopes are summed, and the resulting envelope auto-correlation is calculated. Thus, we have a self-correlation function that reveals the various periodicities of the signal envelope through its dominant peaks. Finally, the beat histogram is formed by the accumulation of all peaks in the sound file. Note that when there is a strong PIH in the signal (usually in strong-beat signals), the histogram peaks will be higher [7].

In this study, six features of the beat histogram were calculated. Algorithms for computational calculations are available in the Essentia library [15] and have the following nomenclatures and definitions:

- First Peak BPM (BH-p1-BPM): value of the highest peak in BPM.
- First Peak Weight (BH-p1-weight): weight of the highest peak.
- First Peak Spread (BH-p1-spread): spread of the highest peak.
- Second Peak BPM (BH-p2-BPM): value of the second-highest peak in BPM.
- Second Peak Weight (BH-p2-weight): weight of the second-highest peak
- Second Peak Spread (BH-p2-spread): spread of the second-highest peak.

### Schematic explanation of the artificial neural networks

As commented, we have performed the machine learning and classification with supervised artificial neural networks (SANN), using the WEKA multilayer perceptron algorithm. The activation function used was the sigmoid $f\left(x\right)=\frac{e^{x}}{1+e^{x}}$. The attribute vector is formed by the following characteristics extracted from each audio signal:

- Audio Signal Visibility Descriptors—ASVD: *k*, *Q*, *N*_*c*_, and Δ
- Audio Signal Processing Descriptors—ASPD: MFCCs, Loud, SFlux, Onset, ZeroCr, and CDyn
- Beat Histogram—BH:bh-p1, bh-p1-spread, bh-p1-weight, bh-p2, bh-p2-spread, and bh-p2-weigh

In the output layer are the musical genres.

#### Scenario 1

**Fig 13:**
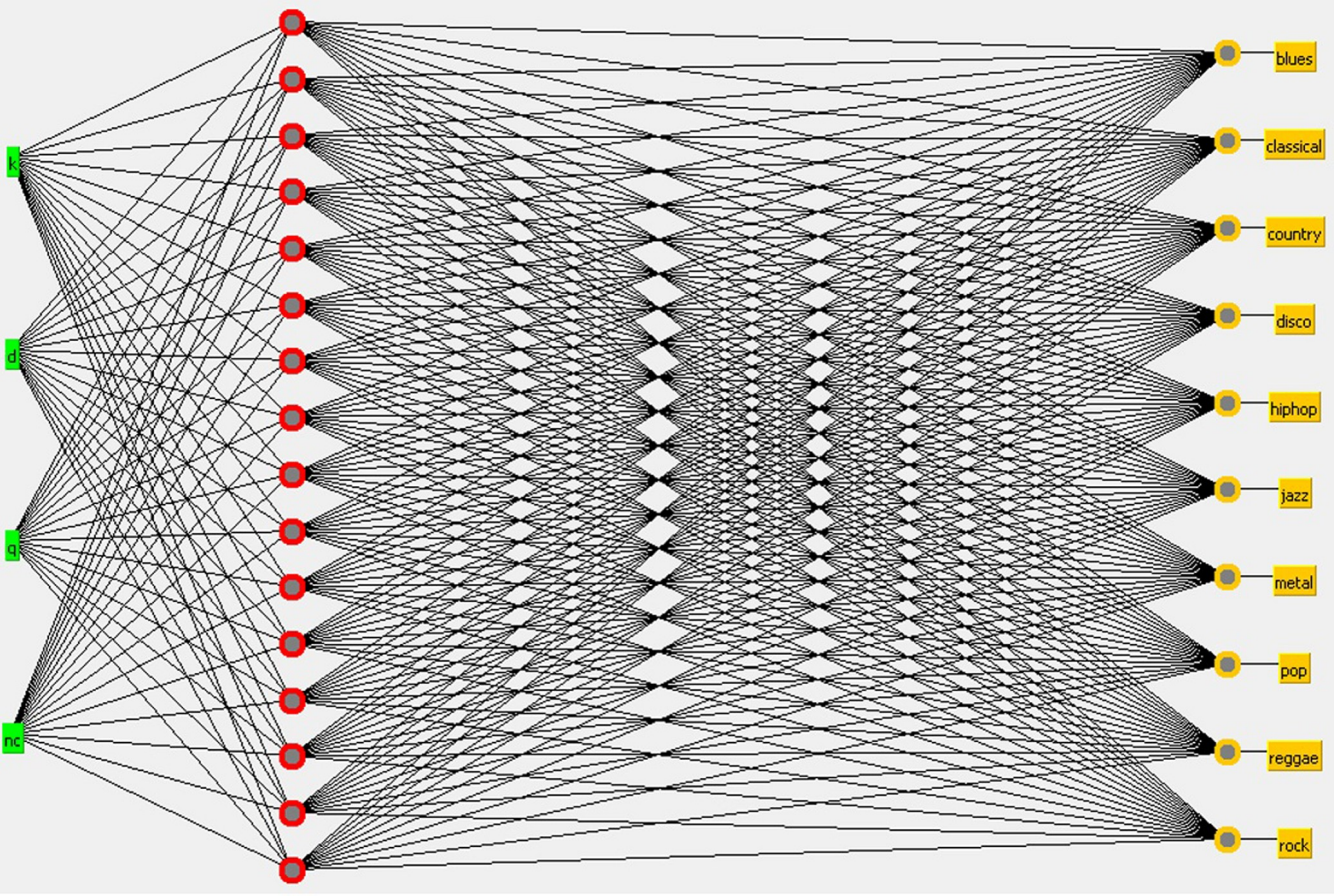
SANN with a hidden layer of 16 neurons considering ASVD at the input layer.

**Fig 14:**
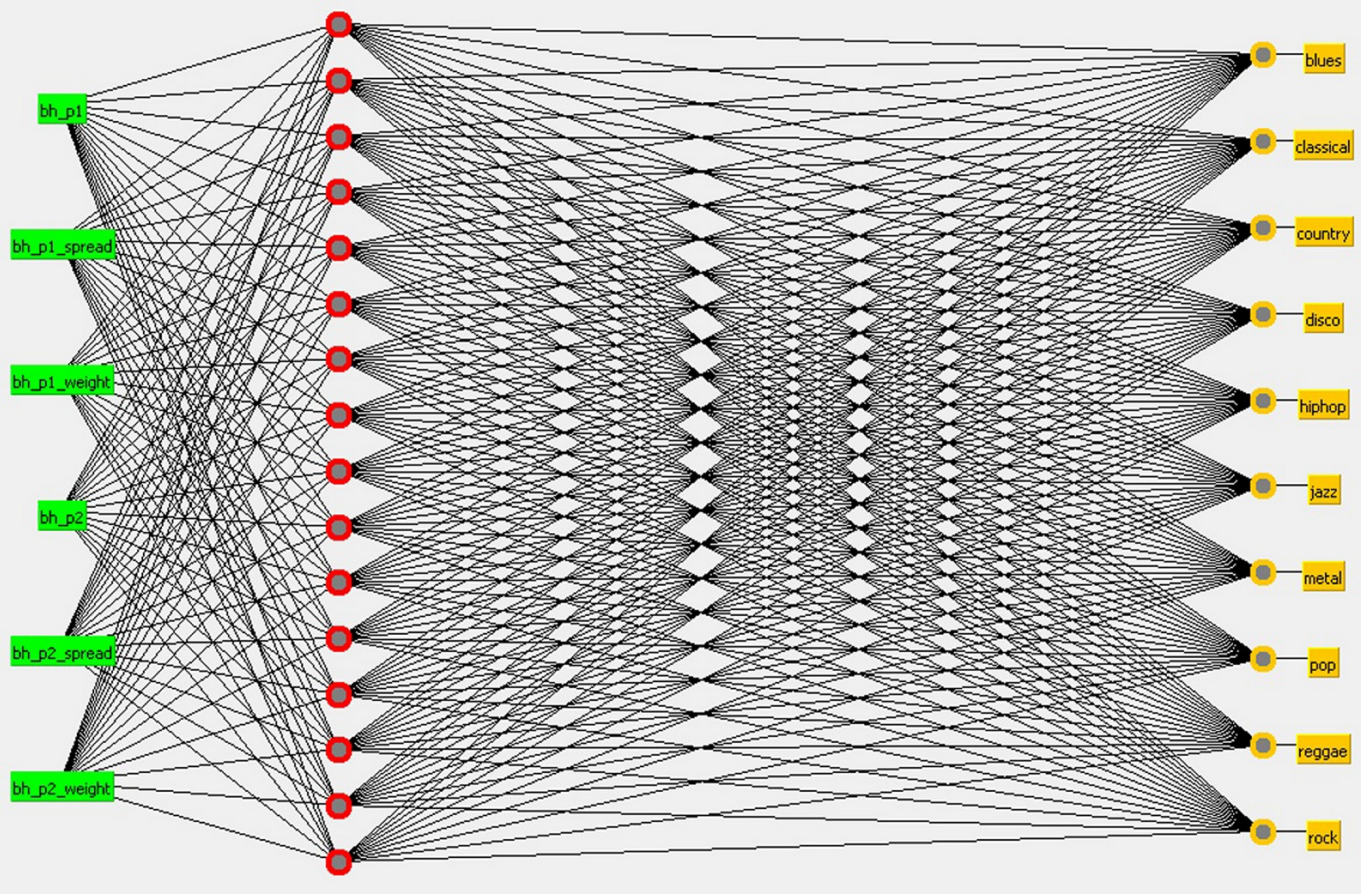
SANN with a hidden layer of 16 neurons considering BH at the input layer.

The SANN that showed the best results were:

- SANN with a hidden layer of 16 neurons using the Audio Signal Visibility Descriptors (Fig 13).
- SANN with a hidden layer of 16 neurons using the Beat Histogram (Fig 14).

#### Scenario 2

**Fig 15:**
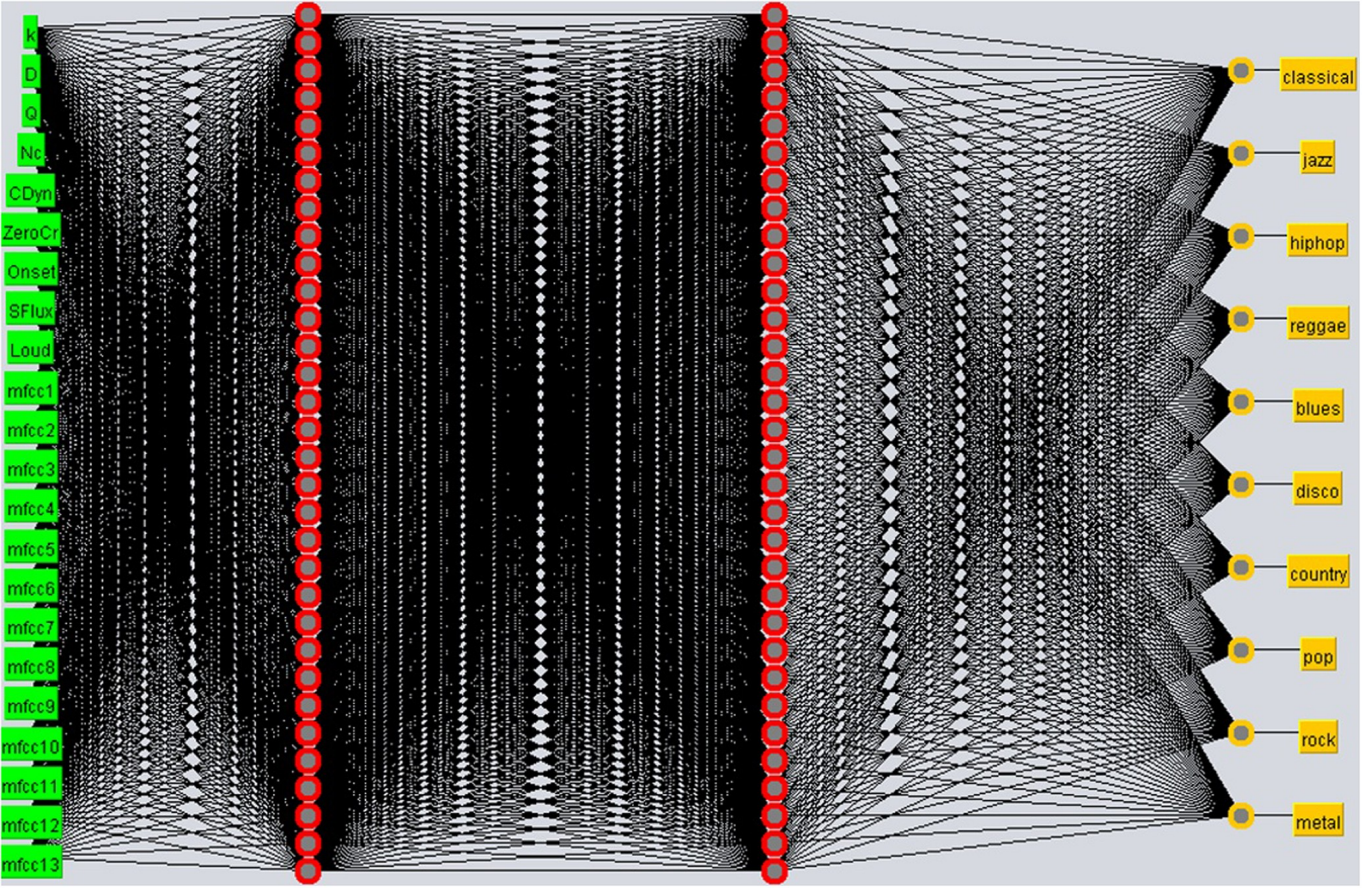
SANN with two hidden layers of 32 neurons and ASVD + ASPD at the input layer.

**Fig 16:**
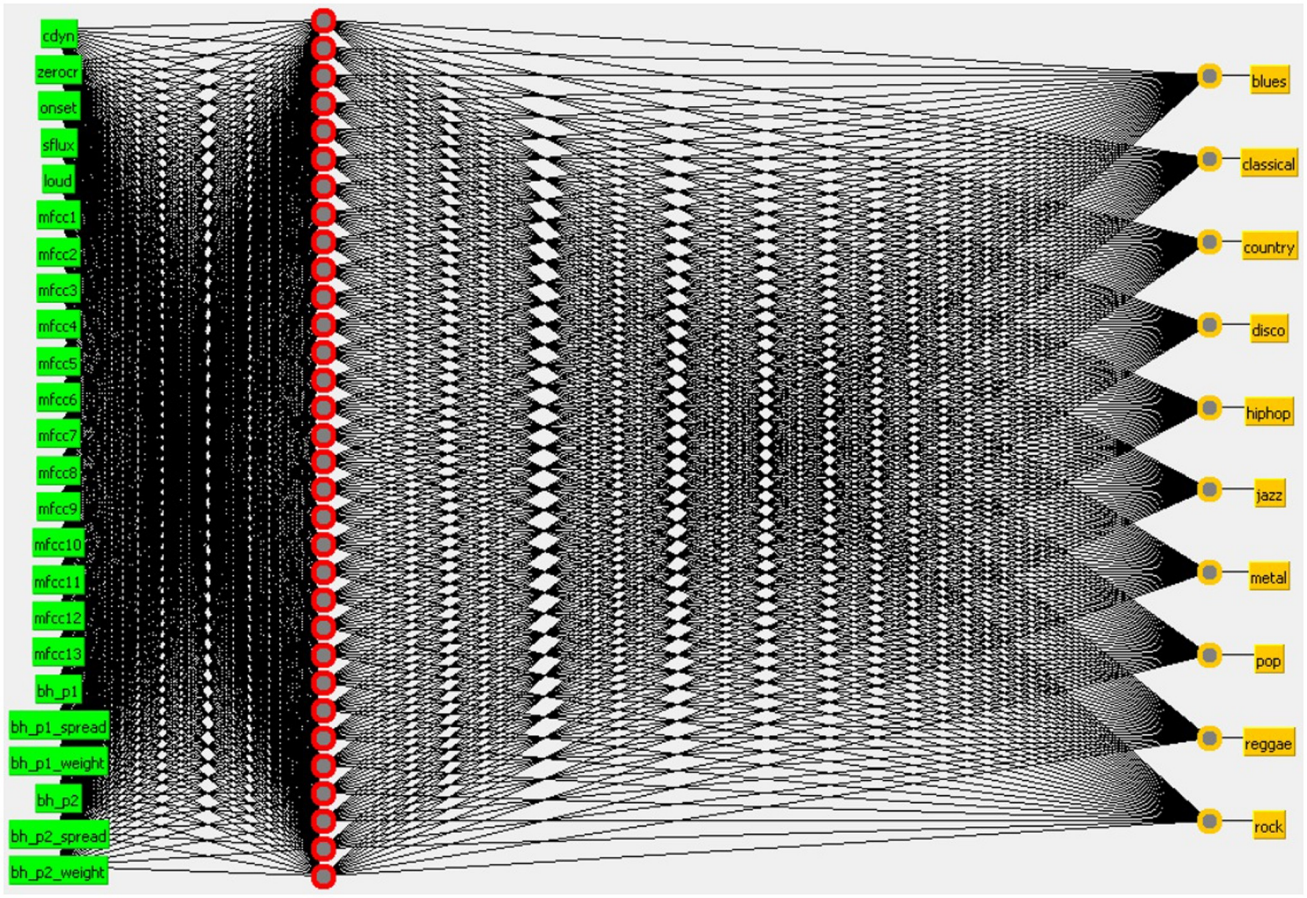
SANN with a hidden layer of 32 neurons and BH + ASPD at the input layer.

The best SANN for the second scenario were:

- SANN with two hidden layers of 32 neurons each network. The attributes used in the input layer were ASVD + ASPD (Fig 15).
- SANN with a hidden layer of 32 neurons and Beat Histogram + Audio Signal Processing Descriptors at the input layer (Fig 16).
